# Specialist Community Nurses: A Critical Analysis of Their Role in the Management of Long-Term Conditions

**DOI:** 10.3390/ijerph6102550

**Published:** 2009-09-29

**Authors:** Gretl A. McHugh, Maria Horne, Karen I. Chalmers, Karen A. Luker

**Affiliations:** School of Nursing, Midwifery & Social Work, University of Manchester, Jean McFarlane Building, Oxford Road, Manchester M13 9PL, UK; E-Mails: maria.horne@manchester.ac.uk (M.H.); karen.chalmers@manchester.ac.uk (K.I.C.); karen.luker@manchester.ac.uk (K.A.L.)

**Keywords:** public health, community nursing, long-term conditions

## Abstract

The aim of this narrative review is to identify strategies in use by specialist community and public health nurses in the prevention, care and management of individuals with long-term conditions, specifically chronic obstructive pulmonary disease (COPD) and musculoskeletal disorders. These conditions have been selected as they are highly prevalent; a burden on health services globally and a major public health issue. From a UK policy perspective, specialist community nurses have been placed at the forefront of taking a lead role in the coordination and delivery of more responsive services for individuals with long-term conditions; whether this has been an effective use of skills and resource is questionable. We systematically searched relevant databases between 1999–2009 to identify interventions used by specialist community nurses and critically appraised the studies. This review reports on impact and value of interventions used by specialist community nurses in the prevention and management of COPD and musculoskeletal conditions, and makes recommendations for improving services.

## 1. Introduction

The increase in prevalence of long-term conditions (LTCs), largely due to the rising elderly population and lifestyle behaviors, is a major public health issue. Globally, the challenge for governments is finding effective health care solutions to manage the rising burden of chronic conditions [1]. In the United Kingdom (UK), there is a drive by the government to manage more effectively individuals with long-term conditions in primary care. This includes emphasizing self-care with patients becoming experts at managing their own condition [2].

The National Health Service (NHS) Improvement Plan [3] sets out the government’s strategy for improving care of people with long-term conditions by moving towards a patient-centered approach. It is recommended that care should be focused in primary care settings but with improved partnerships and communications across all health and social care agencies. The burden on the UK health care system in both primary and secondary care is high. It is estimated that 30% of individuals who report having a long-term condition (LTC), accounts for 52% of all GP appointments, 65% of all outpatient appointments and 72% of inpatient days [4]. Exacerbations of conditions, such as respiratory diseases often lead to hospitalization of individuals, which has economic implications for the NHS.

In order to manage the increasing demands on the health service, the NHS has increased the role that nurses play in the management of individuals with LTCs [5–7]. Practice nurses who work in general practices under the auspices of a general practitioner have had a role in patient care and treatment of individuals with LTCs. Previously, in primary care, community nurses, such as district nurses have had a role in assessment and co-ordination of the health and social care needs of older people. District nurses have two years of nursing experience and have undergone specialist practitioner training. District nurses regularly provide nursing care for individuals with chronic health problems, usually in their own homes or in residential care. As the needs of some patients with LTCs became more complex and with the drive to reducing hospitalization, it was envisaged that nurses working in the community with advanced practice knowledge could play a key role in managing people with complex nursing care needs. The NHS initiated a ‘community matron’ role whereby nurses may become the key worker for patients with long-term health problems. In addition, the case manager role was developed; case managers are usually community trained nurses working under the auspice of the community matron. The community matrons have advanced practice skills (at Master’s level), prescribing rights and knowledge of chronic disease management and their role is to improve the quality of care people with chronic disease receive. It was also envisaged that other nurses at the forefront of community care (including health visitors and public health nurses) could play a role in health promotion and the prevention of further ill health. The public health agenda in the UK is focused on the improvement of health and well being in the population, and this, in part will be achieved by ensuring evidence based health promotion and, treatment and care services of high quality [8]. The role of community nurses are integral to this goal.

Despite government recommendations and the development of more specialist community nursing roles, there appears to be little evidence of their involvement in the management of individuals with LTCs [9–10]. The involvement of community nurses and specialist nurses was minimal in a study of the management of care of individuals with end-stage lower limb osteoarthritis (OA) [10]. A UK national survey of COPD specialist nurse services in the community found little service provision on chronic disease management by specialist/respiratory nurses as well as a ‘mismatch’ between what services were provided for patients with COPD and the existing evidence around effectiveness [11]. In addition, there is little documentation of the interventions or approaches specialist community nurses use in their day-to-day practice or how effective these strategies are in improving patient outcomes.

In order to understand the role of specialist community nurses in the management of long-term conditions, we selected two exemplars, COPD and musculoskeletal conditions, to identify specific research evidence on the effectiveness of community nurses’ interventions in caring for and managing individuals with these two LTCs.

## 2. Methods and Data Sources

### 2.1. Definition

For the purposes of this paper, ‘specialist community nurses’ refers to community nurses with specialist training who work within the community and primary care, providing nursing care in patients’ homes or community. They include district nurses, community matrons, case managers, health visitors and public health specialists. Other nurses working in primary care have been referred to and include ‘specialist respiratory nurses’ who work across hospital and community settings to care for patients with COPD and other respiratory conditions; and ‘practice nurses’ who work in primary care under the auspices of a general practitioner.

### 2.2. Aim

The aim of this review was: (1) to identify strategies used by specialist community nurses in the prevention, care and management of individuals with long-term conditions, specifically COPD and musculoskeletal conditions, and (2) to document the effectiveness of the specialist community nurse role in improving patient outcomes.

### 2.3. Narrative Review

We elected to carry out a narrative review of the literature. This approach to reviews aims to ‘summarize, explain and interpret evidence on a particular topic/question’ using qualitative and/or quantitative evidence [12]. We considered this approach would be appropriate given the aims of the review, the broad range of literature that we were examining and, from the initial scoping of the literature, the likelihood of finding few intervention studies for critique and synthesis.

### 2.4. Types of Studies

All study designs were acceptable for inclusion in the review including randomized clinical trials (RCTs), quasi-experimental studies, pre-post intervention designs (observational studies), descriptive studies and qualitative research studies.

### 2.5. Inclusion/Exclusion Criteria

Inclusion criteria included: studies in which participants were adults (age 18 or older); the intervention was based in the community (or with a significant component in the community); the intervention was evaluated; the intervention was carried out (at least in part) by a specialist community nurse (as defined above) and the intervention was directed to patients with COPD or musculoskeletal disorders. The time frame for inclusion was 1999 to 2009 inclusive and all publications were written in English.

### 2.6. Data Sources

Key databases were searched and included: Medline, Embase, British Nursing Index, CINAHL and all Evidence Based Medicine Reviews (which include Cochrane Database Systematic Reviews; ACP Journal Club; Database of Abstracts of Reviews of Effects (DARE); Clinical Controlled Trials Register (CCTR), Cochrane Methodology Register (CMR), Health Technology Assessment (HTA), and NHS Economic Evaluation Database (NHSEED)). Reference lists from articles/reviews were also searched. The search was limited to the years 1999 to 2009. Only English language studies were included. The rationale for this inclusion was the availability of English language journals for international researchers to access for publication as well as the cost of accurate translation.

The search terms and related terms used in this review were: community, nurses, community nurses, community matron, case manager, health visitor, public health nursing, case management, long-term conditions, chronic obstructive pulmonary disease (COPD), musculoskeletal, nurse-led interventions and primary care.

### 2.7. Search Methods, Study Selection and Quality Assessment

Initially, we considered for inclusion all studies on COPD or musculoskeletal conditions and nurses (including limited related terms), but on conducting the search several thousand titles and abstracts were retrieved. This was narrowed down by using the terms ‘community’ and ‘primary care’ The purpose of examining the literature with this broad range of nursing roles and titles was to identify studies, including international studies, which may be relevant to our review even though the terms the authors used to describe the nursing role may not have precisely mirrored our definition of ‘community nurses’.

In total, six systematic reviews and nine empirical studies were included in the review. The results of the searches were reviewed by two authors who obtained full reports of potentially eligible papers. Studies were assessed for inclusion and relevant content independently reviewed by three authors. The fourth author re-assessed the assignment of the papers to confirm the reliability of assessment and other details. We used tools from the Critical Appraisal Skills Programme (CASP) for assessing the quality of the included studies [13].

## 3. Results and Discussion

### 3.1. Community Nursing Interventions with COPD Patients

The text below highlights the type and breadth of the work being done in caring for COPD patients in the home and the role of the nurse in leading these interventions (referred to as ‘lead nurse’ in the text). Six reviews and four additional empirical studies were identified. Most of the studies did not have a clear definition of the nursing role and little emphasis on what we are defining as a ‘lead role’. However, they provide helpful information on the nursing role in the community with this chronically ill patient group. The main foci of the studies were outreach care in the home, early discharge/hospital-at-home, case management and promotion of patient self-care or elements of these models. See Table 1 for specific details of the reviews and individual studies.

A systematic review of studies (n = 4) of hospital outreach programs using randomized controlled trials (RCTs), of respiratory nurses visiting patients in their homes, has shown no benefit to severely obstructed COPD patients (on the variables of lung function, exercise tolerance; health related quality of life (HRQL) of patients and/or carers, and reduction of mortality and hospital utilization) [14]. However, mortality was improved in moderately obstructed patients. The nurses provided support, education, monitoring and liaison with physicians. Few details were provided, but the nurses appeared to be hospital based specialists in respiratory nursing and not community trained nurses.

The most convincing work on the provision of specialist respiratory nursing care in the home was a systematic review of RCT studies of management of acute exacerbations in the home through early discharge or hospital-at-home programs (n = 7) [15]. The home based care was seen as equivalent to hospital care in terms of patient outcomes (hospital readmissions and mortality). Although few details were provided about the interventions, it appears there was daily monitoring of patients in the home and ready access to specialist medical care, as needed. It is interesting that both experimental and control patients and carers (when asked) stated a preference for home based care. Although the term ‘nurse led’ was not used in these studies, from the descriptions of the care, the role appears fairly congruent with the ‘nurse led’ role.

The effectiveness of innovations in nurse-led chronic disease management for patients with moderate and severe COPD was assessed in a systematic view of nine RCTs [16]. Only three studies met the time criterion (used in our paper) and home care site [17–20], although in one [19] most, but not all, care professionals were nurses. No studies specifically used specialist community nurses although the authors did report that all interventions appeared to be variations on a case study model. The interventions, in most instances, included discharge planning, home visiting, education and support, smoking cessation guidance (where relevant), ongoing assessment, referral/coordination by the nurse, clinical support for the nurse and in a few cases patient education for early recognition of signs and symptoms of exacerbations [18] and fitness advice [17]. Similar to most of the findings reported above, there were no differences between groups on long-term follow-up including mortality, health related quality of life, psychological well being, disability or pulmonary functioning. From this review, there is little robust evidence to support nurse management of chronic disease services for patients in the community with moderate or severe COPD.

Similar results were found in a review of self management educational interventions [21]. Of the identified 14 randomized and one non-randomized studies, there were no significant differences in number of exacerbations, Emergency Room visits, lung function, exercise capacity, and days lost from work between groups. However, the studies showed a significant and clinically relevant reduction in hospitalizations, a small but significant improvement in dyspnea, and improved HRQoL. The authors conclude that data are still insufficient to formulate clear recommendations regarding the form and contents of self management education programs and the need for more large RCTs with a long-term follow-up, before more conclusions can be drawn. The study settings included primary care, hospital and community. As was identified in many of the above studies, there was little documentation of the personnel who delivered the intervention.

Assessment of the best practice evidence for nursing care of COPD patients was examined using the NICE (2004) guidelines [22]. The focus was on key nursing interventions in studies of patients with moderate to severe COPD during a five year period (2000–2005). Twenty-three studies and three systematic reviews (as noted above, [15,17,21]) were identified. The interventions in the studies were classified as smoking cessation, dyspnea management, exercise, hospital-at-home, palliative care and telephone follow-up. As noted above, in many of the studies, it was difficult to sort out if the interventions were carried out by nurses or other health care personnel or just what the nursing role was [23,24]. In one qualitative study of home based palliative care [25], a few (n = 3) patients and carers had access to respiratory specialist nurses; those who did reported positively on the service. It is of note that in one study [26], the nursing intervention was delivered not directly to the patients but nursing home staff using a care protocol which included medication, diet and exercise. The community nursing intervention had no impact on functional and respiratory outcomes or on hospital service utilization.

In a recent narrative review (n = 35), the needs of carers who provide physical care to family members with COPD was assessed including identifying interventions professionals use to support carers in their care-giving role [27]. This review included randomized controlled trials to evaluate patient outcomes of hospital-at-home/early discharge programs and, as noted above, these studies generally reported no negative outcomes for the experimental patients and in some cases, improved outcomes. Other studies were of case management, self–care or similar home care models with a strong focus on improving self management and coordination of care in the home. Similar to the findings noted above, most of these studies examined medical and service utilization outcomes usually with no positive outcomes. The interventions included patient education, assessment and follow-up, but in most cases, few details were provided and the type of nursing care was rarely described in much or any detail. It was also rare that information about patients’ or families’ needs were included in the interventions.

In addition to the studies in the reviews noted above, four additional studies were identified. In a postal study of COPD specialist nurse provision in England and Wales, the number and type of services were identified and compared with the existing evidence of effectiveness [11]. Of the 234 COPD specialist nurse services identified, 71% involved solely chronic COPD management and 17% provided solely acute care services (primarily early discharge rather than hospital at home services). The authors point out the considerable mismatch between the existing evidence of effectiveness of services and provision of services for patients with COPD.

Of the three additional studies identified, findings were fairly similar to those previously reported. No differences in hospitalization re-admission rates or in exacerbations between groups were found in patients discharged from a pulmonary rehabilitation program following hospitalization for an acute exacerbation, although control patients experienced significant higher mortality and higher contacts with providers [28]. The telephone contacts and home visits were provided by specialist respiratory nurses. In addition to monitoring patients’ symptoms, the nurses reinforced advice concerning treatments and lifestyle changes (smoking cessation if relevant, exercise), self management as well as provided patients with written advice on their treatments.

Two international studies also addressed nursing care to COPD patients in the community [29,30]. In a project in Hong Kong, Kwok and colleagues evaluated the prevention of hospital readmissions of older patients with COPD using a RCT design. Post-hospital discharge follow-up by the clinical nurse specialist (CNS) had no measurable benefit on hospital readmission rates, length of hospital stay, or functional or psychological outcomes for older Hong Kong patients [29]. The CNS intervention entailed a specific nursing protocol with close liaison with the designated physicians and continuity of care and the nurses were experienced community health nurses with additional training in respiratory and geriatric care. Benefit was found in a 10 year follow-up study of Italian COPD patients receiving long-term oxygen therapy [30]. Patients receiving the home care program had fewer exacerbations per year and increased survival. While a respiratory nurse was involved in the home follow-up in this study, in addition to clinic services, the nursing role was not described. In summary, while there was benefit to patients from participation in this program, there was little light shed on the contribution of the nursing role to the wellbeing of the patients.

### 3.2. Community Nursing Interventions with Patients with Musculoskeletal Conditions

We did not identify any systematic, narrative and other literature reviews which focused on interventions by specialist community nurses (as defined above) relating to musculoskeletal conditions (e.g. osteoarthritis, rheumatoid arthritis). Most of the reviews focused on interventions provided by secondary care nurses or primary-care led interventions by other health professionals e.g. physiotherapists or general practitioners (GPs). We did identify five empirical studies (three RCTs, one survey and a qualitative study) relating to community nurses’ involvement in the care of patients with musculoskeletal conditions. Table 2 provides an appraisal of these studies.

In the UK, there has been a drive towards developing community-based musculoskeletal services so as to improve access and service delivery for individuals with musculoskeletal conditions. It was envisaged that nurses might play a key role in the delivery of these services. However, a national survey found that three quarters of community musculoskeletal services were led by a physiotherapist or a general practitioner (GP) [31]. There appeared little involvement by specialist community nurses. The study also found there was a lack of monitoring of outcomes and service delivery standards by some of these services.

Despite the emphasis on self management initiatives and community nurses’ potential role with patients with long-term conditions, few studies were identified of community nurses working with patients with musculoskeletal disorders. Victor *et al.* conducted a RCT on the effectiveness of a primary care based nurse-led education program in the UK for people with OA of the knee [32]. This nurse-led intervention consisted of a home visit and four one hour teaching sessions. They found a lack of benefit of the program with little clinical outcome differences in depression, pain or physical ability and in knowledge about OA. Another RCT conducted in The Netherlands also concluded that the nurse-based intervention of education and self management of OA symptoms with older patients did not improve mobility, self-reported function or use of health care resources [33]. The role of homecare nurses in identifying health problems and subsequently providing advice and referrals to manage problems such as, musculoskeletal conditions, has been reported as effective [34]. The complete results from this RCT on the effects on health status and care utilization of visits by home care nurses have not been reported, but from the 160 participants, 95 (63%) were found to have musculoskeletal problems and visits by nurses were recognized as being of value [34,35].

In the final study, qualitative methods were used to explore the involvement of practice nurses and GPs in primary care in patients with OA. Practice nurses reported little involvement in diagnosing and treating OA [36]. Barriers to increased involvement by practice nurses were the lack of knowledge about the disease and treatment. Yet, the patients who were interviewed required information specifically on treatment options and how to manage pain and disability. This study has highlighted that some nurses lack sufficient educational knowledge to specifically manage individuals with musculoskeletal conditions and this may be one reason for the lack of nursing involvement in with patients with OA.

### 3.3. Summary

Taking two exemplars to investigate the effectiveness of specialist community nurses’ interventions in the care and management of long-term conditions has shown that there is a lack of nurse-led initiatives. In patients with COPD, there was evidence of the effectiveness of some interventions carried out by nurses, and in some cases, specialist respiratory nurses in the community, particularly in relation to hospital at home/early discharge roles. In the nurse-led studies of case management or programs to support self-care, findings were mixed. Although the role of the community nurses was often poorly described, it appears to include assessment and monitoring, direct care, patient education, coordination of services, and facilitating ready access to specialist medical care, when needed. These components are all part of the role of the specialist community nurse (and also part of any community nursing role).

In patients with musculoskeletal conditions, there were few studies and little support for the effectiveness of nurse-led interventions in the community for patients with musculoskeletal conditions. The nurse-led education programs did not appear to be effective as reported by two RCTs [32,33]. This may be due to the structure and content and length of delivery time of the nurse-led educational intervention. Overall, there was not much evidence of the effectiveness of the specialist community nurses from the literature reviewed.

### 3.4. Discussion

This narrative review has identified systematic literature and narrative reviews and empirical studies which have evaluated the effectiveness of strategies used by specialist community and public health nurses in the prevention, care and management of individuals with long-term conditions, specifically chronic obstructive pulmonary disease (COPD) and musculoskeletal conditions. Limitations of this narrative review include the focus on English language studies only and the review period from 1999 to 2009. There may have been other studies, which did address specialist community nursing roles that we did not include.

The findings from this review indicate that there is insufficient evidence on the effectiveness of the specialist community nursing roles. This finding stems from problems of documentation in many of the studies, as well as the actual outcomes of the existing empirical work. In many of the studies, there is a lack of specificity as to what types of nurses are providing the intervention. This is more than a matter of semantics; it likely reflects differences in the educational background, professional experiences, degree of focus on the individual patient versus groups of patients and the health care system, and the degree of specialization of the nurses who were participating in the study interventions. In many cases, it was difficult to sort out just what was meant by the terms used to describe the nursing role (i.e., community nurse, home-care nurse, family nurse, respiratory nurse, outreach nurse, etc.). Despite this limitation, as much as possible, we attempted to provide detail on the interventions so that we could assess the degree to which the intervention is similar/not similar to the role of the specialist community nurse. In addition, many of the reviews and studies had inadequate detail on the actual intervention provided to patients. Similar issues have been identified in other studies of nursing care making it difficult to evaluate the contribution of nursing roles and expertise [37].

Overall, there was a paucity of research evidence around the involvement of specialist community nurses in providing services or interventions to patients for both LTCs. The exception was studies of hospital at home or early discharge care of patients with COPD. In these cases, care was deemed ‘equivalent’ in most studies. These interventions appeared to be provided by specialist respiratory nurses. In two of the osteoarthritis (OA) studies, there was no support for an education nurse-led intervention for patients with OA [32,33]. This lack of specificity and clarity in both the role and the intervention make it difficult to uncover the elements of the intervention which may or may not have contributed to the lack of positive outcomes in most studies. It is not clear if the difference in the nurse led care for patients with COPD was the influence of training and specialist skills that the respiratory nurses have. In the studies of patients with OA, the nurses were not specialist musculoskeletal nurses providing the intervention but nurses working in primary care. As noted above, the lack of detail on the intervention, including the skill set of the nurses may ‘mask’ the essential elements of an effective intervention.

The prevalence of musculoskeletal conditions and COPD is set to rise necessitating the need for high quality, coordinated and effective service provision to meet the needs of individuals with these long-term conditions. There is extensive evidence that patients with musculoskeletal conditions, such as OA require improvement in their management as patients report reduced physical functioning and high levels of pain [10,38]. Similar findings of lack of self-care knowledge and behaviors are also noted in COPD patients [39]. Yet, there are few studies with well developed interventions by community nurses, which could assist these patients to manage their conditions more effectively.

One Canadian study looking at nurses’ role in pain management for individuals with long-term problems found that nurses provided little guidance in pain management, prescribing or adjusting pain medications. Numerous barriers contributed to the nurses lack of pain management such as, lack of knowledge in assessing pain and poor collaboration with physicians involved in the patients’ care [40]. Similar barriers have been identified with COPD patients. Few interventions were identified in a systematic review of interventions to assist COPD patients to reduce anxiety and panic, a frequent problem occurring during an exacerbation which often leads patients to seek out emergency hospital services [41]. If community nurses are to become more involved in the prevention, care and management of individuals with these, and other, long-term conditions, more research is needed to develop effective interventions to assist people to cope with their long-term conditions.

There is also a lack of research evidence around community nurses’ involvement in health promotion or public health initiatives. There are a number of public health initiatives which community nurses could get involved in and yet it appears there is a reluctance to tackle some of the fundamental areas which need to be discussed with patients e.g. smoking cessation and strategies to increase physical activity for the prevention of falls [42,43]. This seems to be an important gap in community nursing practice.

There have been some local evaluations which support nurse-led community schemes, specifically for managing patients with COPD [44]. These small scale evaluations show promise that community nursing services do provide benefit. However, well designed studies are needed to produce convincing evidence of the effectiveness of these nursing roles.

We know that there is a need in the UK to improve standards of care for people with long-term conditions [45–47]. WHO’s global strategy on care for chronic conditions emphasizes that when patients receive effective treatments, self management support and regular follow-up, they do better [1]. In order for arthritis and COPD to be managed in primary care, individuals need to be educated to be active partners in their care; and diagnosis, treatment and review need to be carried out by practitioners with adequate knowledge and skills [46,48]. It can be argued that generic community nurses who visit patients at home may require more education and knowledge in order to play a more active and effective role in the care and management of individuals with some long-term conditions. Wilson *et al.* (2006) found that apart from nurse specialists, the majority of nurses (including community nurses) were limited in facilitating self management in patients [49]. Expert patients programs and other self management programs have been shown to be effective in some patient populations [50,51]; these are lay tutor led self management initiatives. It has been suggested that nurses are not comfortable with expert patients [49]. If community nurses are hesitant to work in partnership with others and to assert a role in promoting self management with long-term care patients in the community, they may find that this role is promoted with other groups, such as lay carers. There is evidence that lay carers achieved similar outcomes in education for self management in patients with asthma [52]. Clearly, nurses in all roles in the community must position themselves as key stakeholders in caring for patients in the community and support this position with knowledge, skill and evidence of effectiveness.

Changes are also needed in the delivery of health care systems. The UK National Health Service (NHS) is trying to delivery a more effective and quality service delivery for individuals with long-term conditions. The future vision is for high quality community care that encourages patients to manage their own care and increase the range of support provided for people with long-term conditions [53]. People with long-term conditions are asking for more services to be delivered safely and effectively in the community or at home [4]. The WHO in their global strategy on chronic care management outlines that organized systems of care, and not just individual health workers are essential for producing positive outcomes [1]. Changing just one part of the health service, such as promoting more case management is not sufficient without a change in how care is organized including the relationships between continuing care and acute care [54]. Health care providers, for example, do not routinely ask patients their smoking status and desire to stop smoking despite long standing evidence that brief assessment and interventions are effective in smoking cessation [42]. Assessment protocols in hospitals and community health settings could be structured to facilitate addressing these important self-care and public health problems.

## 4. Conclusions

Community nurses are in a key position to promote health through their access to individuals, families and communities. However, they need to strengthen their nursing and public health roles to plan and deliver more public health strategies to reduce the burden of musculoskeletal diseases and COPD in the future. There needs to be a coordinated effort and more partnership working to change systems of care. There also needs to be more research to determine the effectiveness of community nurse-led interventions for the management of COPD and musculoskeletal conditions.

## Figures and Tables

**Table 1.** Systematic Reviews and Empirical Studies of Interventions with Patients with COPD.

**Systematic Reviews**						
**Authors, Date, Location**	**Study Design**	**Sample**	**Intervention**	**Outcomes**	**Main Findings**	**Comments**
Smith *et al.* 2001, UK	4 RCTs	Severe and moderate COPD	Outreach nurse in home	Mortality, service utilization	No differences in severe COPD patients, benefit with moderate COPD patients; considerable missing data in some studies	Nurse provided support, education, monitoring and liaison with physicians
Ram *et al.* 2004, Scotland	7 RCTs	COPD patients presenting at ER with exacerbation	Patients randomized to home support by a specialist respiratory nurse or regular hospital care	Hospital re-admissions, mortality	No significant differences in outcomes (i.e. home care had not negative outcomes). Patients and carers in both groups preferred care at home	Specialist respiratory nurses had frequent visits &/or telephone contact with patients and direct access to medical advice
Taylor *et al.* 2005, UK	9 RCTs	Patients were inpatient, outpatient & community based with moderate or severe COPD	Intervention was led by nurses & included case management, education, support, coordination of services	Mortality, health related quality of life, psychological well being, disability or pulmonary functioning	There were no differences between groups on long-term follow-up including mortality, health related quality of life, psychological well being, disability or pulmonary functioning	Studies were led, coordinated or delivered by nurses. Little robust evidence to support nurse management of chronic disease services for community patients
Tinker & While 2006, UK	23 studies & 3 systematic reviews, all designs included.	Patients with moderate to severe COPD	Focus on key interventions as recommended by NICE guidelines (smoking cessation, dyspnea management, exercise, hospital-at-home, palliative care and telephone follow-up)	Outcomes relevant to type of study, i.e. hospital-at-home, dyspnea reduction, smoking cessation.	5 relevant studies indicating no adverse effect of hospital-at-home (4) and some positive benefit of symptom management and service utilization	Interventions were delivered by specialist respiratory nurses (in 4 studies) and community nurses (1 study)
Effing *et al.*2007, The Netherlands	14 RCTs 1 non-random trial	Clinical diagnosis of COPD, patients with asthma were excluded	COPD education and/or self-treatment guidelines (i.e. an action plan).	Health related quality of life (HRQoL), symptoms, number and severity of exacerbations, self-treatment of exacerbations, hospital admission, ER visits, days lost from work, lung function, exercise capacity	The studies showed a significant and clinically relevant reduction in hospitalizations, a small but significant improvement in dyspnoea and improved HRQoL. No effects were found in number of exacerbations, ER-visits, lung function, exercise capacity, and days lost from work.	Difficult to identify if interventions took place in outpatient department or home and role of community nurse (if any)
Caress *et al.* 2009, UK	35 studies, all designs	COPD patients	Interventions of early discharge/hospital at home (HH) case management and self–care or similar models.	Range of outcomes including mortality, service utilization, re-admission rates and other variables such as quality of life (QoL), knowledge & satisfaction with care	Early discharge/hospital at home – no negative outcomes & in some cases positive outcomes. Case management/self-care – QoL, knowledge, satisfaction with care increased. Mixed findings on medical outcomes	Few details on the type of nursing care was provided in most reports
**Empirical Studies**						
**Authors, Date, Location**	**Study Design**	**Sample**	**Intervention**	**Outcomes**	**Main Findings**	**Comments**
Candy *et al.* 2007, UK	Postal survey of respiratory healthcare workers and findings compared with 2 systematic reviews	234 COPD specialist nursing services	Type of service documented	Most services addressed chronic disease management	Majority of services (71%) addressed chronic disease management for which there is little empirical evidence of effectiveness	This is the first survey of specialist nurse service provision
Kwok *et al..* 2007, Hong Kong	RCT	77 CNS 80 controls	Post discharge home visits and access to community nurse specialist, telephone follow-up & medical support from designated physician	Prevention of readmission, length of stay, physical functioning & psychological variable	No significant differences between groups	Home care delivered by community nurse specialist
Sridhar *et al.* 2008, UK	RCT	122 COPD patients	Nurse-led intermediate care program. pulmonary including rehabilitation & self management education, written personalized COPD action plan, monthly telephone calls, 3 monthly home visits by a specialist nurse for two years	Hospital readmission rates, unscheduled primary care visits	No differences in re-admission rates or in exacerbations between groups. Higher mortality in control group	Home care delivered by specialist nurse
Rizzi *et al..* 2009, Italy	Non-randomized 2 group design	108 home care (HC) 109 standard care	Outpatient clinical and functional assessments every six months by a specialist team including a pneumonologist, respiratory nurse, home evaluations of the patients at the request of patients and/or caregivers, and respiratory therapist visits (every two to three months or more frequently), liaison with the patient’s general practitioner	10 year follow-up of mortality, exacerbations, hospital and intensive care use	HC group reduced lower mortality, exacerbations, hospital and intensive care use	Respiratory nurse was part of domiciliary team

**Table 2.** Empirical Studies of Interventions with Patients with Musculoskeletal Conditions.

**Authors, Date, Location**	**Study Design**	**Sample**	**Intervention**	**Outcomes**	**Main Findings**	**Comments**
Roberts *et al*. 2003, UK	Postal survey of health professionals	461 primary care organizations (5 professional roles in each of the organizations)	Type of community based musculoskeletal services provided	Organizations had one or more musculoskeletal services in their community	71% provided one or more musculoskeletal services; Services led largely by physiotherapists (47%) and GPs (31%). Community nurses part of the service profile but role not identified. Also lack of evidence to support effectiveness of their services	Community nurses did not appear to have a role in community based musculoskeletal services
Victor *et al*. 2005, UK	RCT	22 GP practices	Primary care based patient education program – efficacy of nurse-led education program – program home visit and four 1 hour teaching sessions (intervention); education booklet only (control)	Quality of life - using SF-36; OA measure, arthritis helplessness index and patient knowledge questionnaire	No differences in groups in depression, OA knowledge or pain and physical ability	Lack of benefit of a nurse led primary care-based patient education program
Rosemann *et al*. 2006, Germany	Qualitative: semi-structured interviews	20 patients with OA; 20 GPs and 20 practice nurses	Hypothesis that patients lack information on disease, medication, and possible approaches	Evidence base on non-surgical treatment options; attitudes towards large involvement of practice nurse in care of patients with OA	Practice nurses had little involvement in diagnosing OA and in treatment; barriers against involvement: lack of knowledge about disease and treatment. Patients feel that pain and disability are addressed in-adequately by GPs	Patients require more information on how to manage pain and disability
Nicholaides-Bouman *et al*. 2007, The Netherlands (Process evaluation) Nicholaides-Bouman *et a.l* 2004, (Trial design details)	RCT	160 older people with health problems (95 (63%) with musculoskeletal problems) 170 controls received usual care	8 nurse visits over 18 months; visits in intervention group received visits from same nurse every 2 months. Visiting protocol and older assessment system and EasyCare Questionnaire used to detect further problems	Process evaluation of home visiting program; participants’ experiences with visits. Effect on health status and care utilization (outcome from RCT not yet complete)	151 received visits. 95 participants had musculoskeletal problems, from these there were 220 (14%) problems, and 249 (14%) were provided with interventions. Health visiting program by home nurses is appreciated by older people as many problems are detected and interventions such as advice or referrals were provided	Papers reports on process evaluation, trial not yet published so no results on health status and care utilization
Wetzels *et al.* 2008, The Netherlands	RCT	51 patients with mild hip or knee OA to intervention group; 53 to control group	Support patients’ self management of OA symptoms using a practice-based nurse. Nurse aimed to change lifestyle behavior, by improving mobility and physical functioning	Patients’ mobility (Timed up and GO Test); Patient Reported Functioning (Arthritis specific scale – Dutch AIMS2 SF)	Nurse-based intervention did not improve older OA patient’s mobility and functional status or use of health care resources.	Nurses provided home visits and telephone follow–up. Sample was small. Supports findings of Victor et al’s (2005) study that nurse-led education program with OA did not benefit these patients
